# Clinical impact of restless legs syndrome in patients with migraine: a 12-year, single-center, longitudinal study

**DOI:** 10.1007/s41105-024-00547-8

**Published:** 2024-08-05

**Authors:** Keisuke Suzuki, Shiho Suzuki, Yasuo Haruyama, Hiroaki Fujita, Koichi Hirata

**Affiliations:** 1 Department of Neurology, Dokkyo Medical University, 880 Kitakobayashi, Mibu, Shimotsuga, Tochigi 321-0293 Japan; 2 Integrated Research Faculty for Advanced Medical Sciences, Dokkyo Medical University, Mibu, Japan

**Keywords:** Migraine, Restless legs syndrome, Headache-related disability

## Abstract

Although many studies have indicated a significant association between migraine and restless legs syndrome (RLS), few long-term longitudinal studies have examined RLS in patients with migraine. We conducted a single-center, 12-year, longitudinal study of migraine patients and assessed whether RLS was present in 2010, 2017, or 2022 to evaluate its associations with clinical factors. Headache-related disability was assessed using the Migraine Disability Assessment (MIDAS). Sleep quality, daytime sleepiness, and depressive symptoms were assessed using the Pittsburgh Sleep Quality Index (PSQI), the Epworth Sleepiness Scale (ESS) and the Beck Depression Inventory-II (BDI-II), respectively. Of the 262 patients included at baseline (2010), 101 were available after 7 years (2017), and 74 were available after 12 years (2022). The RLS incidence rates were 13.7%, 20.8%, and 24.3% in 2010, 2017, and 2022, respectively. The RLS severity score did not significantly differ among the three time points. The persistent RLS group, defined as those who were positive for RLS at the last evaluation in addition to the first and/or second evaluations, had significantly higher MIDAS, BDI-II, PSQI and ESS scores than did the never RLS group, defined as those who did not exhibit RLS at any of the three time points. Our 12-year longitudinal study revealed significant impacts of RLS on the burden of patients with migraine.

## Introduction

Migraine is a common disabling neurological disorder that affects approximately 15% of the general population worldwide [1]. Patients with migraine typically experience recurrent episodes of severe headache, as well as vomiting, photophobia, phonophobia, and a variety of other physical and psychological signs and symptoms [2]. The coexistence of sleep disturbances and migraine has been attributed to underlying hypothalamic dysfunction [3]. Sleep disturbances increase the risk of transition from episodic migraine to chronic migraine and are associated with poor outcomes in patients with chronic migraine [4], emphasizing the importance of managing sleep disturbances in migraine patients in clinical practice. Restless legs syndrome (RLS) is a sleep-related movement disorder characterized by an urge to move the legs, accompanied by an uncomfortable leg sensation, which occurs mainly at night and interferes with sleep. RLS is a common comorbidity of migraine and causes insomnia and daytime dysfunction [5, 6]. In patients with migraine, comorbid RLS has been reported to affect depressive symptoms, sleep quality, and headache-related disability [7–9]. Premonitory symptoms such as yawning, nausea, somnolence, and food craving are more common in migraine patients with RLS than in those without RLS, suggesting the involvement of dysfunction in the hypothalamic dopaminergic system [10]. In a study in the general population, RLS was significantly associated with increased age [11]; however, long-term longitudinal data on the prevalence of RLS in migraine patients are lacking. In the present research, we aimed to investigate changes in RLS incidence and the impact of RLS on clinical factors in migraine patients through a 12-year follow-up study.

## Methods

**Fig. 1:**
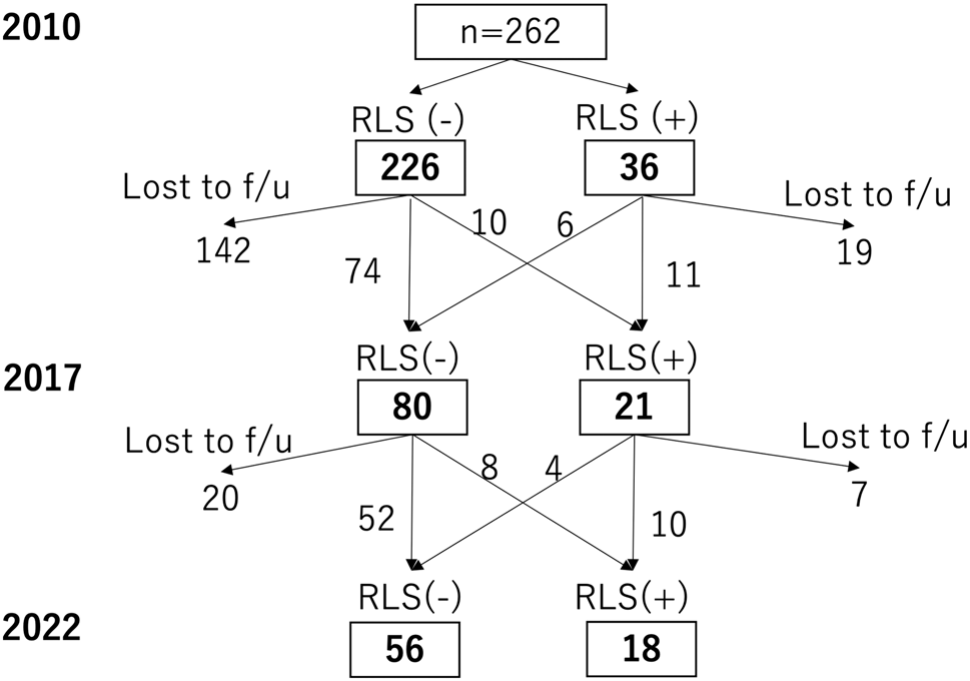
Changes in RLS status over time. f/u = follow-up

We conducted a single-center longitudinal study. The study was carried out in accordance with the guidelines of the Declaration of Helsinki and was approved by the Institutional Review Board of Dokkyo Medical University Hospital. All participants provided written informed consent to participate in the study. Our university hospital is located in Tochigi Prefecture, which is in the Kanto region known as the Greater Tokyo Area. Our outpatient clinics consist of adult general neurology and specialized outpatient clinics, such as dementia and headache clinics. A referral letter is recommended when visiting our headache outpatient clinic but is not mandatory. In 2010, at our single center, 262 migraine patients (age, 38.2 ± 13.0 years; 47 men and 215 women; aura, 25.6%; chronic migraine, 24.8%) were evaluated for RLS; in 2017, 101 patients were available for follow-up, and in 2022, 74 patients were available for follow-up (Fig. 1). Migraine was diagnosed by a headache specialist according to the International Classification of Headache Disorders, 2nd edition (ICHD-2) in 2010 [12] and was confirmed according to the ICHD-3 in 2022 [13].

RLS was diagnosed by a neurologist at three time points according to established criteria [14]. Specifically, a diagnosis of RLS required four key features during the past year: (1) an urge to move the legs, usually accompanied by uncomfortable and unpleasant sensations in the legs; (2) occurrence or worsening of symptoms at rest or with inactivity; (3) partial or total relief of symptoms by movement; and (4) symptoms that occur or worsen only during the evening or night. After careful consideration of the differential diagnosis and exclusion of RLS mimics, a definite diagnosis of RLS was made. The International Restless Legs Syndrome Study Group Rating Scale (IRLS) was used to assess the severity of symptoms [15]. Headache-related disability during the past 3 months was assessed using the Migraine Disability Assessment (MIDAS) [16]. Sleep quality and daytime sleepiness were assessed using the Pittsburgh Sleep Quality Index (PSQI) [17] and Epworth Sleepiness Scale (ESS) [18], respectively. Depressive symptoms were assessed using the Beck Depression Inventory-II (BDI-II) [19]. The 74 patients who were available for follow-up in 2022 were also classified into three groups according to RLS status: never RLS (no RLS at any of the three time points), intermittent RLS (positive for RLS at any of the three time points), and persistent RLS (positive for RLS at the last evaluation in 2022 as well as at the first and/or second evaluation).

### Statistical analysis

Chi-square tests were used to compare categorical variables among groups. One-way analysis of variance (ANOVA) followed by the Bonferroni test was used to compare continuous variables among groups. The PSQI, ESS, BDI-II, and MIDAS scores according to RLS status (never RLS, intermittent RLS, or persistent RLS) were compared using analysis of covariance (ANCOVA) adjusted for age and sex with the Bonferroni post hoc test. Multinomial logistic regression analysis was performed to determine the associations between RLS status (never RLS, intermittent RLS, or persistent RLS) and four clinical parameters (MIDAS, ESS, PSQI, and BDI-II scores), adjusted for sex and age. A two-sided p value < 0.05 was considered to indicate statistical significance. IBM SPSS version 29 (IBM SPSS, Tokyo, Japan) was used for all the statistical analyses.

## Results

**Fig. 2:**
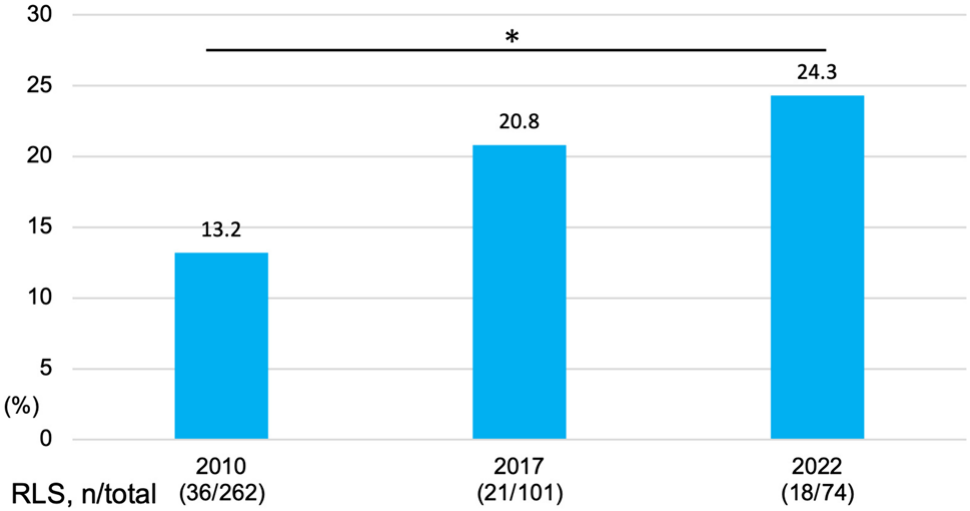
Changes in RLS incidence over time in patients with migraine. Chi-square tests were used. *p < 0.05

**Fig. 3:**
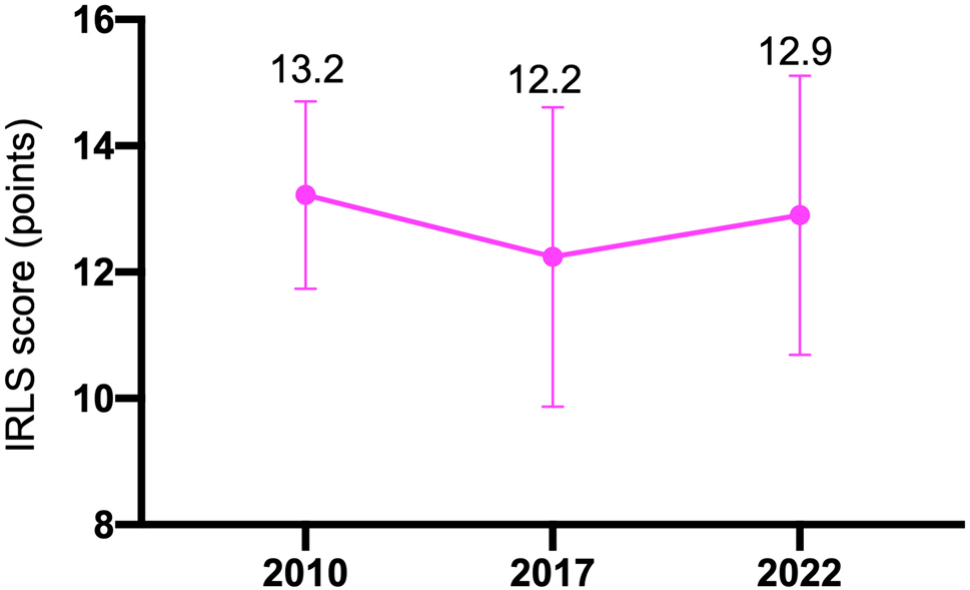
Changes in RLS severity scores over time. ANOVA was used. p = 0.933. The circles indicate the means, and the error bars indicate standard errors of the means

**Fig. 4:**
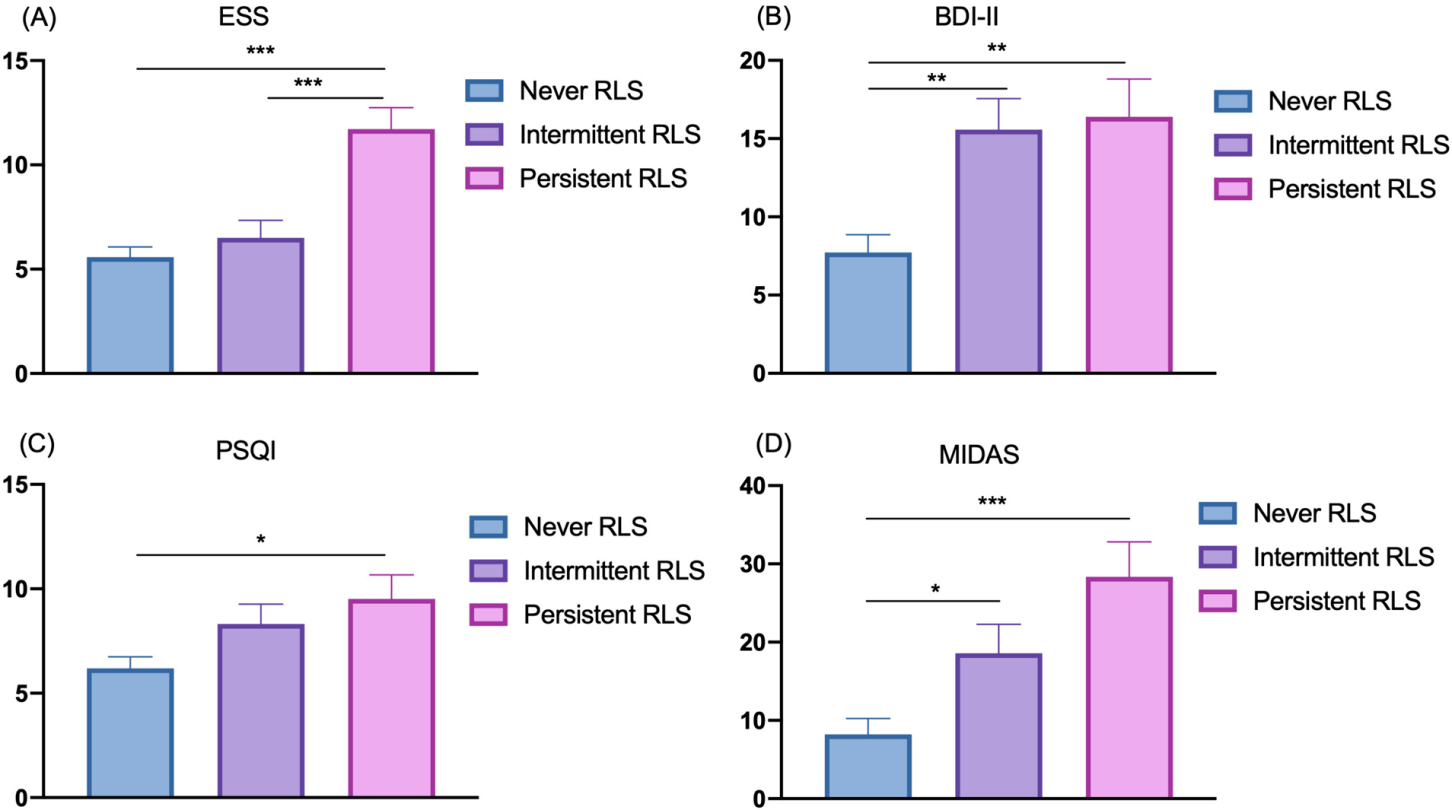
Changes in four clinical factors according to RLS status. **A** ESS, **B** BDI-II, **C** PSQI, and **D** MIDAS scores were compared among the never RLS, intermittent RLS and persistent RLS groups. The scores on the vertical axis indicate the mean values, and the error bars indicate the standard error of the means. ANCOVA adjusted for age and sex was used, with the Bonferroni test. *p < 0.05; **p < 0.01; ***p < 0.001

Figure 2 shows the changes in the RLS incidence rates at the three time points. The RLS incidence rates were 13.7%, 20.8%, and 24.3% in 2010, 2017, and 2022, respectively, and the rate significantly increased in 2022 from 2010. The IRLS score did not differ among the three time points (Fig. 3). The MIDAS, BDI-II, ESS and PSQI scores according to RLS status (never RLS, intermittent RLS, or persistent RLS), analyzed with ANCOVA adjusted for age and sex, are shown in Fig. 4. The persistent RLS group had significantly higher MIDAS, BDI-II, PSQI and ESS scores than did the never RLS group.

The values of the clinical variables in 2022 according to RLS status (never RLS, n = 49; intermittent RLS, n = 15; and persistent RLS, n = 10) are shown in Table 1. No patient had iron deficiency anemia, end-stage renal disease, fibromyalgia or epilepsy. Psychiatric diseases such as anxiety and depression were found in 2 (4.1%), 3 (20.0%) and 2 (20.0%) patients in the never RLS group, intermittent RLS group and persistent RLS group, respectively. In the never RLS group, there were fewer psychiatric comorbidities (4.1%), but most of the 21 patients (42.9%) treated with antidepressants were prescribed them as migraine prophylaxis. No patient took antipsychotics. In the persistent RLS group, 30% of the patients were treated with dopamine agonists, and in the intermittent RLS group 6.7% were treated with alpha 2 delta ligands. The persistent RLS group had higher MIDAS, ESS, PSQI and BDI-II scores than did the RLS group. The BDI-II score was greater in the intermittent RLS group than in the never RLS group. There was a difference in the ESS score between the intermittent and persistent RLS groups. Multinomial logistic regression analysis, controlling for age and sex, revealed that the MIDAS, ESS, PSQI, and BDI-II scores were significantly greater in the persistent RLS group than in the never RLS group (the reference group), and the MIDAS, PSQI and BDI-II scores were significantly greater in the intermittent RLS group than in the never RLS group (Table 2).

**Table 1:** Clinical variables in 2022 according to the RLS status of migraine patients

	Never RLS	Intermittent RLS	Persistent RLS	p value^a^
N (M/F)	49 (6/43)	15 (1/14)	10 (1/9)	0.828
Age (years)	53.4 ± 11.0	55.4 ± 9.8	52.2 ± 14.3	0.762
Disease duration (years)	34.9 ± 11.6	34.3 ± 12.8	36.5 ± 12.7	0.903
Caffeine, n (%)	46 (93.9)	13 (86.7)	7 (70.0)	0.081
Alcohol, n (%)	26 (53.1)	8 (53.3)	5 (50.0)	0.983
Smoking, n (%)	6 (12.2)	3 (20.0)	3 (30.0)	0.346
*Comorbidities, n (%)*				
Psychiatric diseases	2 (4.1)	3 (20.0)	2 (20.0)	0.086
Heart disease	2 (4.1)	0 (0.0)	0 (0.0)	0.592
Hypertension	5 (10.2)	0 (0.0)	2 (20.0)	0.235
Dyslipidemia	5 (10.2)	0 (0.0)	1 (10.0)	0.436
Diabetes mellitus	2 (4.1)	0 (0.0)	1 (10.0)	0.462
*Treatment, n (%)*				
Antidepressants	21 (42.9)	3 (20.0)	2 (20.0)	0.150
Antiepileptic drugs	15 (30.6)	5 (33.3)	3 (30.0)	0.977
Hypnotics	1 (2.0)	2 (13.3)	1 (10.0)	0.188
*RLS treatment, n (%)*				
Alpha 2 delta ligands	0 (0.0)	1 (6.7)	0 (0.0)	0.136
Dopamine agonists	0 (0.0)	0 (0.0)	3 (30.0)	< 0.001
MIDAS score	8.2 ± 10.9¶	18.4 ± 20.4	28.8 ± 20.0	< 0.001
ESS score	5.6 ± 3.1¶	6.3 ± 4.5§	11.8 ± 1.2	< 0.001
PSQI score	6.2 ± 3.4¶	8.2 ± 4.4	9.4 ± 3.8	0.016
BDI-II score	7.7 ± 5.2¶	15.5 ± 11.4#	16.5 ± 9.6	< 0.001

*BDI-II* Beck Depression Inventory-II, *ESS* Epworth Sleepiness Scale, *MIDAS* Migraine Disability Assessment, *PSQI* Pittsburgh Sleep Quality Index, *RLS* restless legs syndrome

^a^Using a chi-square test or ANOVA

^¶^p < 0.05 compared to persistent RLS

^§^p < 0.05 compared to persistent RLS

^#^p < 0.05 compared to never RLS

ANOVA followed by Bonferroni test

**Table 2:** Multinomial logistic regression analysis of the associations of RLS status with four clinical parameters

	cOR	95% CI	p value	aOR	95% CI	p value
*MIDAS score*						
Never RLS	1.0 (ref)			1.0 (ref)		
Intermittent RLS	1.062	1.012–1.116	0.015	1.072	1.015–1.131	0.012
Persistent RLS	1.086	1.031–1.144	0.002	1.096	1.035–1.162	0.002
*ESS score*						
Never RLS	1.0 (ref)			1.0 (ref)		
Intermittent RLS	1.071	0.895–1.281	0.454	1.099	0.915–1.320	0.311
Persistent RLS	1.602	1.233–2.083	< 0.001	1.617	1.242–2.105	< 0.001
*PSQI score*						
Never RLS	1.0 (ref)			1.0 (ref)		
Intermittent RLS	1.170	0.991–1.382	0.064	1.195	1.003–1.424	0.046
Persistent RLS	1.265	1.049–1.525	0.014	1.273	1.049–1.546	0.015
*BDI-II score*						
Never RLS	1.0 (ref)			1.0 (ref)		
Intermittent RLS	1.179	1.063–1.308	0.002	1.181	1.063–1.312	0.002
Persistent RLS	1.189	1.065–1.327	0.002	1.187	1.063–1.326	0.002

*BDI-II* Beck Depression Inventory-II, *ESS* Epworth Sleepiness Scale, *MIDAS* Migraine Disability Assessment, *PSQI* Pittsburgh Sleep Quality Index, *RLS* restless legs syndrome, *cOR* crude odds ratio, *aOR* odds ratio adjusted for sex and age

## Discussion

In the present study, we observed changes in RLS incidence during a single-center, long-term, follow-up study of patients with migraine. The incidence of RLS at 3 time points spanning 12 years ranged from 13.7 to 24.3%, with an increase in incidence at the last assessment compared with the initial assessment. RLS severity was assessed at 3 time points in patients with comorbid migraine and RLS; severity did not significantly differ over time. In a study of the general population, the association of RLS with migraine was significant in patients aged < 50 years and in those with migraine headaches ≥ 1 day per month [20]. In a large cross-sectional study, RLS complication rates and RLS severity scores were greater in migraine patients than in healthy controls [21]. However, no prospective study of migraine patients has reported trends in the severity or prevalence of RLS; therefore, we believe that this study represents an important contribution.

Compared with the never RLS group, the persistent RLS group had significantly worse headache-related disability severity, depressive symptoms, insomnia and daytime sleepiness. The present findings regarding the impact of RLS on these clinical factors in this longitudinal study extend our previous findings [7]. Consistent with our results, several cross-sectional studies have reported that insomnia, depressive symptoms, and daytime sleepiness are more severe in migraine patients with RLS than in migraine patients without RLS [6, 22, 23]. Since sleep disturbances may be one of the modifiable risk factors for the chronicity and progression of migraine [24, 25], therapeutic intervention for RLS may reduce the severity of headache-related disability and disease progression.

The pathophysiological mechanisms shared by migraine and RLS are thought to involve dopaminergic dysfunction, impaired iron metabolism, and genetic factors [5, 6]. A recent genome-wide analysis revealed two novel loci associated with RLS in patients with migraine in addition to six previously identified RLS risk loci, namely, MEIS1, BTBD9 and PTPRD [26]. Other studies have reported several risk factors for RLS in patients with migraine, such as vitamin D deficiency [27] and pharmacological overload of serotoninergic drugs [28]. In our previous study, however, antidepressant use did not differ between migraine patients with and without RLS [8]. In addition, the patients in the intermittent and persistent RLS groups were less likely to take antidepressants compared to those in the never RLS group, suggesting that antidepressants are unlikely to be involved in inducing RLS symptoms in our study.

The limitations of this study include the lack of follow-up of healthy controls, patient selection bias that patients recruited at university hospitals tend to be refractory, and the sample size, which was initially 262 and was reduced to 74 by the final follow-up. It is possible that patients with less severe migraine at our university hospital were referred to local clinics, leaving patients with severe migraine; this background may have influenced the study results since RLS has been reported to occur more frequently in chronic migraine patients than in episodic migraine patients [29]. Microstructural changes in the basal ganglia in patients with comorbid migraine RLS have been previously described [30]. However, this prospective study did not evaluate functional brain imaging data, so changes in the effects of RLS on the brain structure of migraine patients are unclear. Our data did not include baseline data on body mass index, so the percentage of obesity was unavailable. We did not adjust for comorbidities or medications that may affect RLS status, depressive symptoms, sleep quality, sleepiness or disability because of the small sample size of our study. Further studies with larger sample sizes, adjusting for these factors, are needed to confirm our results.

In conclusion, our 12-year longitudinal study of patients with migraine revealed significant effects of RLS on headache-related disability, insomnia, depressive symptoms, and daytime sleepiness.

## Data Availability

The relevant data are included within the paper, but the data sets from this study are available from the corresponding author upon reasonable request.
